# Emerging Pathogens Pose Inevitable Surprises

**DOI:** 10.3201/eid2902.AC2902

**Published:** 2023-02

**Authors:** Byron Breedlove

**Affiliations:** Centers for Disease Control and Prevention, Atlanta, Georgia, USA

**Keywords:** art science connection, emerging infectious diseases, art and medicine, about the cover, public health, Emerging Pathogens Pose Inevitable Surprises, Jack in the Box, emerging pathogens, AIDS, Ebola, Zika, COVID-19, severe acute respiratory syndrome, Middle East respiratory syndrome, mpox, bacteria, viruses, mice, rodents, zoonoses

**Figure:**
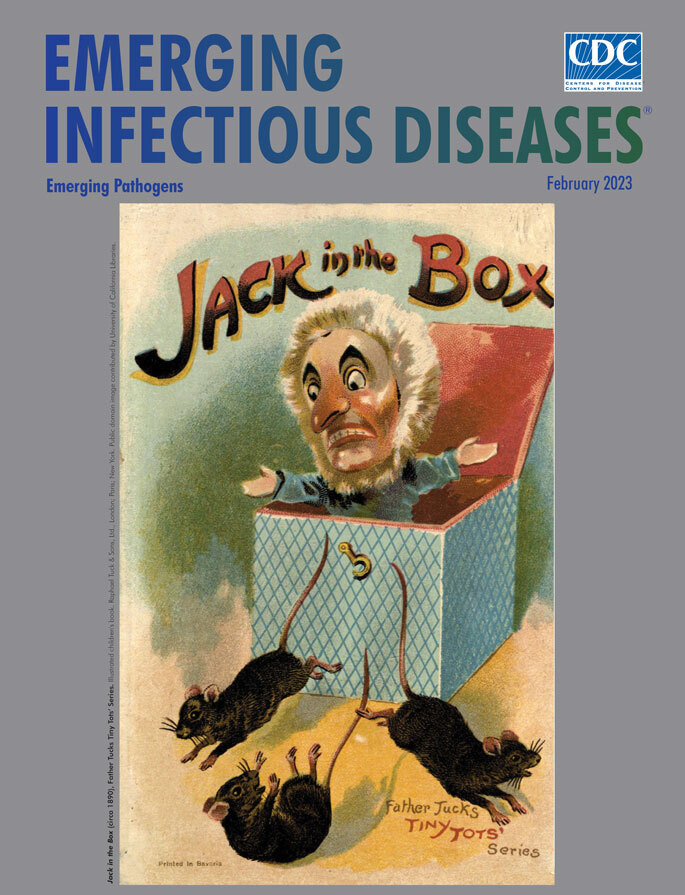
***Jack in the Box* (circa 1890),** **Father Tuck’s Tiny Tots’ Series**. Illustrated children’s book. Raphael Tuck & Sons, Ltd., London; Paris; New York. Public domain image contributed by University of California Libraries.

Anthony Fauci, who retired as head of the National Institute of Allergy and Infectious Diseases, National Institutes of Health, at the end of 2022, wrote that “The emergence of new infections and the reemergence of old ones are largely the result of human interactions with and encroachment on nature. As human societies expand in a progressively interconnected world and the human–animal interface is perturbed, opportunities are created, often aided by climate changes, for unstable infectious agents to emerge, jump species, and in some cases adapt to spread among humans.” 

Factors that can lead to emergence of pathogens also include contaminated food and water, modern agricultural practices, human migration, use and misuse of antimicrobial agents, and lack of public health resources in some areas. In 2013, Morens and Fauci wrote, “The inevitable, but unpredictable, appearance of new infectious diseases has been recognized for millennia, well before the discovery of causative infectious agents. Today, however, despite extraordinary advances in development of countermeasures (diagnostics, therapeutics, and vaccines), the ease of world travel and increased global interdependence have added layers of complexity to containing these infectious diseases that affect not only the health but the economic stability of societies.”

A number of emerging diseases confirm the challenge of predicting when and where an outbreak may occur and how severe and widespread it may be. Examples include the first cases of what would be known as AIDS in the early 1980s, multiple outbreaks of Ebola in parts of Africa, Zika in South and North America, severe acute respiratory syndrome, Middle East respiratory syndrome, and COVID-19. 

Fauci noted, “Today, there is no reason to believe that the threat of emerging infections will diminish, since their underlying causes are present and most likely increasing.” Invariably, public health experts will be surprised by some yet unknown emerging pathogen or disease outbreak. This month’s cover art, an image of a jack in the box from the cover of a children’s book, speaks to the element of surprise. 

Believed to date from the 1500s according to various sources, including the Strong National Museum of Play, this enduring children’s toy is essentially a puppet, clown, or caricature of a person mounted on a spring inside a box. After jostling a latched box or cranking a handle to wind the spring, a child is rewarded with a short tune before the “Jack” suddenly pops open the lid, eliciting a laugh or cry. The Victoria and Albert Museum notes, “Despite their frightening qualities such toys were not only cheap and popular, but also helped to provide children with their first awareness of basic scientific principles.”

This month’s cover image, whose creator is not credited, appeared on the cover of one of the many books from the Father Tuck’s Tiny Tots’ Series (circa 1895), produced by Raphael Tuck & Sons, Ltd., a company that produced children’s books, postcards, greeting cards, puzzles, and various ephemera. The grimacing figure in the image, arms splayed outward, has emerged from its unlatched box and frightened a trio of prowling mice. Rodents, in turn, are known to spread various emerging pathogenic agents and could factor in to the next unexpected disease outbreak. As humans encroach with their machines, the animals run away, but nature will return and perhaps have already left something even more frightening in the animals’ wake. It is not surprising, therefore, that the recommendations from the 1992 Institute of Medicine report *Emerging Infections: Microbial Threats to Health in the United States* underscoring the value of public health surveillance, preparedness, and infrastructure remain relevant and applicable.
